# Linkage of Viral Sequences among HIV-Infected Village Residents in Botswana: Estimation of Linkage Rates in the Presence of Missing Data

**DOI:** 10.1371/journal.pcbi.1003430

**Published:** 2014-01-09

**Authors:** Nicole Bohme Carnegie, Rui Wang, Vladimir Novitsky, Victor De Gruttola

**Affiliations:** 1 Department of Biostatistics, Harvard School of Public Health, Boston, Massachusetts, United States of America; 2 Division of Sleep Medicine, Brigham and Women's Hospital, Boston, Massachusetts, United States of America; 3 Department of Immunology and Infectious Diseases, Harvard School of Public Health, Boston, Massachusetts, United States of America; Imperial College London, United Kingdom

## Abstract

Linkage analysis is useful in investigating disease transmission dynamics and the effect of interventions on them, but estimates of probabilities of linkage between infected people from observed data can be biased downward when missingness is informative. We investigate variation in the rates at which subjects' viral genotypes link across groups defined by viral load (low/high) and antiretroviral treatment (ART) status using blood samples from household surveys in the Northeast sector of Mochudi, Botswana. The probability of obtaining a sequence from a sample varies with viral load; samples with low viral load are harder to amplify. Pairwise genetic distances were estimated from aligned nucleotide sequences of HIV-1C env gp120. It is first shown that the probability that randomly selected sequences are linked can be estimated consistently from observed data. This is then used to develop estimates of the probability that a sequence from one group links to at least one sequence from another group under the assumption of independence across pairs. Furthermore, a resampling approach is developed that accounts for the presence of correlation across pairs, with diagnostics for assessing the reliability of the method. Sequences were obtained for 65% of subjects with high viral load (HVL, n = 117), 54% of subjects with low viral load but not on ART (LVL, n = 180), and 45% of subjects on ART (ART, n = 126). The probability of linkage between two individuals is highest if both have HVL, and lowest if one has LVL and the other has LVL or is on ART. Linkage across groups is high for HVL and lower for LVL and ART. Adjustment for missing data increases the group-wise linkage rates by 40–100%, and changes the relative rates between groups. Bias in inferences regarding HIV viral linkage that arise from differential ability to genotype samples can be reduced by appropriate methods for accommodating missing data.

## Author Summary

The analysis of viral genomes has great potential for investigating transmission of disease, including the identification of risk factors and transmission clusters, and can thereby aid in targeting interventions. To make use of genetic data in this way, it is necessary to make inferences about population-level patterns of viral linkage. As with any rigorous statistical inference from sampled data to a population, it is important to consider the effect of the sampling strategy and the occurrence of missing data on the final inferences made. In this paper we highlight the effects of missing data on the resulting estimates of population level linkage rates and develop methods for adjusting for the presence of missing data. As an example, we consider comparing the rates of linkage of HIV sequences from subjects with high viral load, low viral load, or on antiretroviral treatment, and show that comparative inferences are compromised when adjustment is not made for missing sequences and bias in inferences can be reduced with proper adjustment.

## Introduction

Interest has been growing in the use of viral linkage analysis to investigate disease transmission dynamics and the effect of interventions on them [1]–[8]. To optimize interventions intended to control the HIV epidemic, it will be useful to identify host characteristics (e.g. disease status and demographics) that are associated with high rates of clustered or genetically-linked infections. Many studies attempt to make inferences about linkage patterns in a larger population than that represented by the set of observed viral genetic sequences without considering the effect of sampling or missing data (see, e.g. [5], [9]). However, estimates of probabilities of linkage that ignore the impact of missing data (henceforth referred to as *unadjusted estimators*) can be biased downward. In order to estimate the amount of linkage in communities or compare rates of linkage across groups we must properly account for the presence of missing data.

The work presented here arose from a desire to compare linkage rates between demographic groups found via a household survey from the Mochudi study, an HIV prevention program for Mochudi, Botswana (R01 AI083036; PI: M. Essex; www.aids.harvard.edu/news/spotlight/archives/v6i3_mochudi_project.html). Young males were found to be severely underrepresented, making inferences about linkage involving this group unreliable. As information regarding the size of this subpopulation is available, it is possible to leverage it to improve inferences. This household survey is part of a pilot project leading to a large community-randomized trial, also in Botswana, of a combination HIV prevention intervention, the Botswana Combination Prevention Project (BCPP; U01 GH000447; PIs: M. Essex & V. De Gruttola) [10], [11]. One of the goals of the BCPP study is to leverage viral linkage to understand the patterns of mixing across communities and the relative contributions of within-community and outside-community sources to new infections.

This paper develops estimators for linkage probabilities under the assumption that unobserved sequences are missing at random conditional on observed information. We consider analyses in which linkage is defined by a threshold on the pairwise distance between viral sequences. The choice of the threshold is an important scientific question in the analysis of viral genetic data, but the methods developed here apply regardless of the particular value of the threshold chosen, or can be applied to range of thresholds of interest. We first show that the probability that randomly selected sequences are linked can be estimated unbiasedly from observed data. We then derive an estimate of conditional probabilities of linkage between groups given the existence of a link, and consider estimation of group-level probabilities of linkage. We first develop estimators under the assumption that indicators of linkage are independent across pairs of individuals who may be linked – an assumption that could be appropriate in situations with either a very sparse graph or sparse sampling in the population. We then develop a bootstrap resampling approach that is approximately correct under general assumptions about the structure of correlations of linkage indicators across pairs. Finally, we propose a diagnostic approach for assessing the reliability of the method.

We apply the methods developed to analyses of viral sequences from the northeast sector of the village of Mochudi in Botswana, the site of a pilot study intended to determine the feasibility of testing for HIV infection in a household setting and linking infected subjects to care. Our investigation focuses on assessment of whether rates at which subjects' HIV genotypes link with others depends on ART treatment status and viral load levels (low/high) among the untreated. Such clustering reflects underlying HIV transmission dynamics; a tendency for subjects with high viral load to link more frequently with others might suggest an increased role of subjects with elevated levels of viral replication in HIV transmission. This is also consistent with high viremia in early infection; the contribution of those with elevated viral load to onward spread is difficult to assess in samples of prevalent cases due to the fact that a subject's category varies over time. With high prevalence, however, it is unlikely that a high proportion of subjects in the sample are newly infected; nonetheless, this approach will be particularly useful in the analysis of data from the BCPP, which will identify incident cases and permit comparison of their linkage rates with the groups discussed here.

## Methods

Consider a population of hosts partitioned into 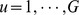 disjoint groups, each of size 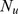. Groups might be defined by demographic characteristics, risk behavior, disease stage, etc. To make our estimates of linkage probability identifiable, we must make a standard assumption that missingness is random conditional on group membership, so the group definitions should include all characteristics relevant to the probability of observation of a sequence from a given host. Suppose that the probability that a sequence from group 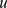 is in our sample, 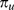, is known. In each group, we thus observe viral sequences for a subset of hosts of size 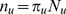. Let 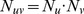 (or 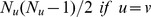) be the number of pairs of sequences between groups 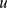 and 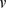. Two sequences (representing two individual hosts) are considered to be linked if the genetic distance between them is less than some threshold value. Let 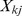 be an indicator for a link between sequences 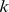 and 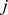.

### Probability of linkage between two individuals

Our first goal is to estimate the probability of linkage between viral sequences from two individuals selected at random from their respective groups, 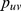. Under the assumption of missingness at random (MAR) conditional on group membership, the 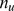 hosts for whom viral sequences are available represent a random sample of the total population of their group, and by extension the observed linkage indicators 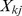 are a random sample of the linkage indicators for the full population. Thus, the Law of Large Numbers tells us that the sample average, 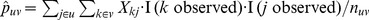, converges to the population mean, 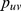.

As a result, under the assumption of MAR conditional on group, it is possible to obtain an unbiased estimate of the probability that a pair of sequences are linked without adjustment for missing data.

### Conditional probability of linkage

One quantity of interest in the analysis of a community randomized trial such as the BCPP is the relative probability that a new infection arises from contact with an infected person from within a community versus from outside the community. Therefore we may wish to estimate the conditional probability, 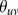, that a pair of sequences 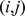 are from groups 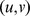, given that 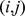 are linked. If missingness is completely at random (unconditional on group), then we can use the observed proportions of links in each group pair 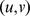, 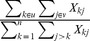, with 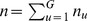, to estimate the conditional probabilities. If missingness is MAR conditional on group, as we assume, this estimate requires adjustment for the differing missingness rates between groups. In a population of size 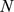, there are 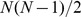 total possible pairs. The probability of linkage for a randomly selected pair is given by
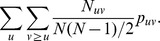
The probability that a randomly selected pair is from groups 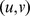 and is linked is
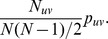
Thus, the conditional probability we desire is
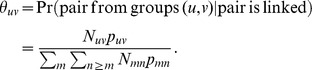
We substitute 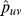 into these formulas to obtain a plug-in estimator 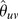 of 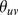. Note that this derivation does not require an assumption of independence, so we can consistently estimate the conditional probability of linkage regardless of the underlying correlations of linkage indicators across pairs.

### Estimation of linkage rates between groups

Based on the results above, we now focus on estimation of the probability that a randomly selected sequence from group 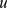 links with at least one sequence from group 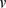 (excluding itself if 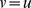), 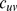. In this case the unadjusted estimate of the probability of linkage between groups will be an underestimate of the true rate: any sequence that does not link with any other in the observed data may in fact link with sequence(s) from the community that were not observed. Thus, the proportion of observed sequences in group 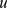 that do not link with any sequence in group 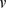 will be higher than the proportion in the population.

For the purposes of exposition, we begin with an assumption of independence among linkage estimators, but we extend to a case with individual-by-group random effects driving the correlations among indicators. This flexible model accounts for correlations due to individual factors – biology, behavior, network position – as well as differential interactions of individuals with different groups.

#### Plug-in estimation under independence

We begin by assuming that indicators 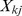 of linkage between a sequence in 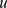 with any sequence in 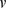 are mutually independent. We wish to estimate
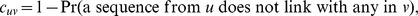
which is 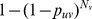 under independence. We obtain a plug-in estimator by substituting the estimate 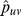 for the true 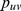, 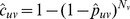. The expected value of this quanitity is not available in closed form, but in general will not be equal to 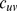. The variance of 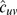, is similarly difficult to write in closed form, but we do know that 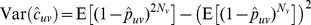. Both values can easily be evaluated numerically.

These results can easily be extended to estimation of the probability that a sequence from group 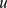 or a set of groups 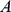 links with at least one sequence in a set of groups 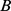 (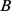 can intersect 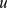 or 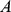, with adjustments to group sizes to exclude self-ties). In the first case, the estimator is 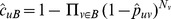, with expected value equal to one minus the product of the expected values of 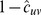 for 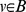 and variance 
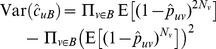
. We estimate linkage between sets of groups 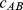 by a weighted average of 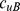 for 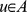, with the weights given by the size of group 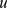 in the population, 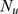. Thus, 
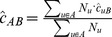
, and the expected value and variance are the appropriate weighted sums of the expected value and variance of the 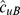.

#### Relaxing independence assumptions: Bootstrap bias estimation

We consider bootstrap estimation of the bias in the unadjusted estimate in order to accommodate deviations from independence among pairs. The development begins by assuming independence and then relaxes this assumption. The expected percent of sampled sequences in group 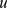 that cluster with at least one observed sequence in group 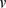 is 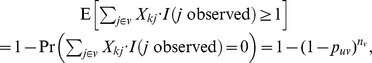 where 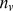 is the number sampled out of a population of size 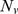 and 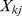 is an indicator for linkage between sequences 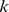 and 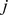. For a sampling probability of 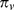, we expect 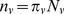. Note that the unadjusted estimate of the probability of no clustering, 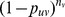, differs from the truth, 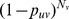 by a ratio of 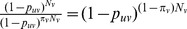. An alternative to direct calculation of the MLE is to estimate this ratio and use it as an adjustment factor to correct the unadjusted estimator.

We can estimate this ratio given the observed data in one of two ways. The first involves taking a subsample with probability 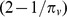 from the observed sample to obtain a sample of approximately 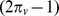 percent of the full population and taking the ratio of the rates of non-linkage in the observed sample and the subsample as the adjustment factor (this gives an exponent of 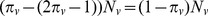). This method is denoted *interval subsampling* because the sampling proportions for the population, observed sample, and bootstrap subsample are at equal intervals. It limits the sizes of samples for which adjustment can be made; a subsample of appropriate size is impossible for 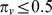, and in practice the bound is higher, as an arbitrarily small subsample will be likely to miss all observed links.

An alternative approach takes a subsample with probability 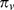 from the observed sample, and uses the ratio of rates as described above raised to a power of 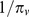 to get an estimate of 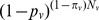. This approach, denoted *proportionate subsampling*, extends the range of sample sizes for which the bootstrap is practical.

#### Exchangeable correlation

Suppose all linkage indicators 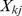 for sequence 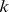 in group 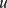 with sequences in group 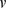 are distributed as exchangeable Bernoulli random variables with probability 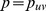 and correlation 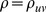. Defining 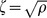, we can express 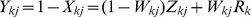, where the 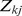 and 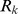 are iid Bern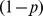 and the 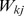 are iid Bern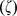 [12]. We find the probability that none of the 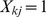 by taking the expectation of 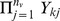. For simplicity, we suppress the subscript 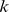 in what follows. In the product 


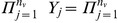

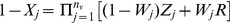
, 

 all terms subscripted by 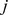 are raised to a power of at most 1 in any element. Since the 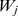, 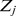 and 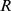 are all mutually independent random variables, the resulting expectation is merely the product of their expectations. Thus we can simplify the expression by replacing 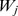 and 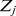 with their respective expected values, 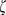 and 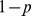, to obtain 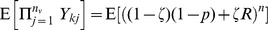. Thus,
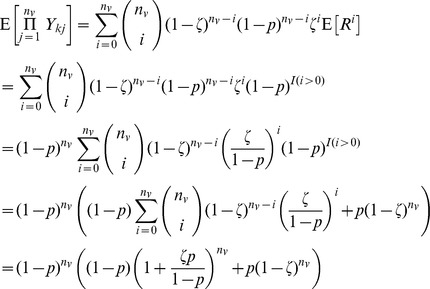


For the values of 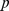, 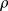 and 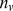 that we are likely to encounter, 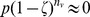. This means that using the methods described for the independence case, we are trying to estimate
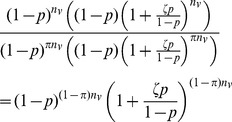
by (interval subsampling)
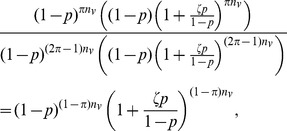
or (proportionate subsampling)
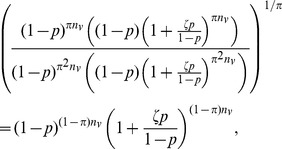
and hence the bootstrap bias correction will be approximately unbiased for 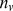 sufficiently large under either interval or proportionate subsampling.

A note on estimating 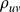: we are assuming that 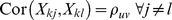, with 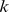 in group 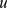 and 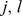 in group 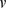 (Note that 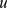 may equal 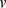 if we are interested in within-group linkage). Under this assumption, the expected number of linked pairs between groups 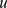 and 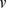 in which 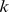 is a participant remains the same as in the independence case. Supposing we have a population of size 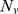 and the probability that a pair is linked is 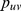, then 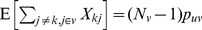, just as if the 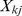 were uncorrelated (note that we are excluding 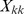 as we do not include self-links). The variance, on the other hand, is affected by the correlation. 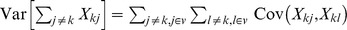. In the independence case, this is merely 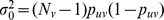, but in the exchangeable case we have 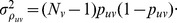

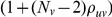
. Given 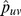, we can thus estimate 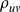 by 
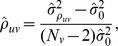
 where 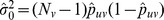 and 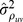 is the empirical variance of the number of links by sequence. Note that for fixed 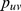, 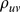 is invariant in 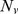, so we can estimate the correlation in the population using the correlation in the sample.

#### Random effects

We can relax the assumption that correlation varies only by group pairing to permit each sequence to have its own baseline correlation with each group (a version of the classic random effects model), by allowing 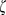 to vary with 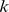 and 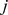. Suppose 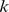 is a member of group 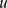 and 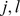 are members of group 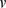, and let 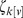 be a baseline propensity of sequence 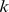 to link with sequences in group 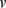. Then the correlation between 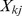 and 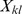 is 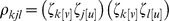 rather than 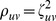. This gives us (after replacing singleton independent random variables with their expected values, as in the development for exchangeability) 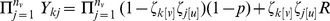, and
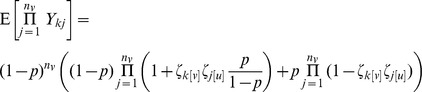
We still expect 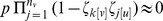 for the sample sizes of interest, so the bootstrap bias correction will be approximately correct as long as
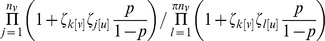
is well approximated by one of the bootstrapped quantities
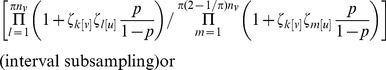

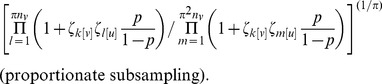
If we assume that the 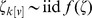 for some distribution 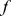, and let 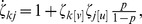 then, for any set of sequences 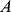, we have
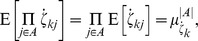
so 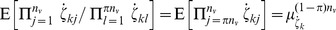. If we use the interval subsampling method described above, then we have
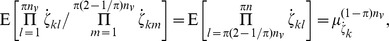
so the bootstrap adjustment is correct in expectation. If, on the other hand, we have a smaller sample and want to use the proportionate subsampling approach, we have
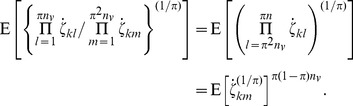
By Jensen's Inequality, 
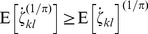
, indicating that the bootstrap method in this case will under-correct, on average.

## Results

As mentioned in the introduction, we apply the methods described above to viral sequences from a household survey in Mochudi, Botswana. HIV-1 subtype C sequences used in this study represent the initial genotyping effort within the Mochudi Prevention Project. Briefly, viral sequences were obtained by nucleic acid extraction from dry blood spots collected during a household survey in Mochudi and two rounds of PCR amplification of HIV-1 env gp120 V1C5 region with primers ED3/ED14 and ED5/ED12 [13] followed by direct sequencing of amplified products as described previously [14]. Sequence contigs were assembled by SeqScape v.2.7 (Applied Biosystems), and generated viral sequences were aligned by Muscle [15], [16]. To prevent and control for contamination, QA/QC procedures were applied routinely during all experimental steps. We generated sequences of the *env* gp120 region from 423 subjects from the first year of the survey (GenBank accession numbers KF374112, KF374117-KF374123, KF374125-KF374132, KF374134-KF374138, KF374141, KF374142, KF374144, KF374147-KF374151, KF374153, KF374156, KF374157, KF374159-KF374161, KF374163-KF374171, KF374174, KF374175, KF374177-KF374181, KF374183, KF374184, KF374186-KF374196, KF374198-KF374217, KF374219-KF374221, KF374223, KF374224, KF374227, KF374230, KF374231, KF374233, KF374234, KF374237-KF374239, KF374241-KF374246, KF374248-KF374250, KF374252, KF374253, KF374255-KF374265, KF374267-KF374271, KF374273, KF374275-KF374280, KF374282-KF374284, KF374287, KF374289, KF374291-KF374307, KF374309-KF374313, KF374315, KF374318-KF374323, KF374325-KF374327, KF374329-KF374332, KF374335, KF374337, KF374339, KF374341-KF374343, KF374345, KF374347, KF374349-KF374354, KF374356-KF374373, KF374376, KF374377, KF374379, KF374380, KF374382-KF374384, KF374387-KF374389, KF374391, KF374392, KF374394, KF374396-KF374402, KF374404, KF374405, KF374407-KF374413, KF374415, KF374417-KF374420, KF374424-KF374426, KF374428-KF374432, KF374434, KF374436-KF374446, KF374448, KF374449, KF374451-KF374454, KF374457-KF374459, KF374462-KF374467, KF374470, KF374471, KF374474-KF374481, KF374484-KF374490, KF374492, KF374494-KF374496, KF374498-KF374502, KF374504-KF374513, KF374518, KF374520-KF374523, KF374525, KF374526, KF374528, KF374531-KF374533, KF374535-KF374540, KF374542, KF374543, KF374546-KF374550, KF374553-KF374555, KF374558, KF374560-KF374565, KF374569-KF374573, KF374575, KF374576, KF374579-KF374581, KF374585, KF374587-KF374598, KF374601, KF374604, KF374606-KF374613, KF374617-KF374620, KF374622, KF374623, KF374626-KF374631, KF374633, KF374634, KF374636, KF374638-KF374640, KF374642, KF374645-KF374652, KF374654-KF374656, KF374658, KF374660, KF374661, KF374663-KF374665, KF374668-KF374678.).

Interest lies in assessing the impact of viral load levels on rates of linkage, but the probability of being able to sequence a sample depends on viral load, given that low VL samples are more difficult to amplify. From the household survey, we retrieved 791 subjects with data on viral load and treatment status, which we divide into three categories: high viral load (HVL, >50 K copies/mL), on antiretroviral treatment (ART), and low viral load (LVL, ≤50 K copies/mL, no ART). We subdivide those with viral load less than 50,000 copies/mL by treatment status because the processes that lead to the lower viral load are likely different for these two groups. At the time of analysis, viral sequences were available for 65% of HVL subjects, 54% of LVL subjects and only 45% of those on ART. The size of the groups also varies, with 23, 42, and 35 percent of the sample being HVL, LVL and ART, respectively.

### Phylogenetic tree simulation

As a first step in validating the performance of the approach, we perform a simulation study applying our methods to data simulated from an evolutionary model. To implement the simulation, we used SeqGen v1.3.2 [17]. We obtained the tree required as input to the program by fitting a maximum likelihood tree to the 423 observed sequences from Mochudi, and parameterized the evolutionary model by fitting the general time-reversible model with gamma distributed rate heterogeneity to those sequences and using the estimated parameters (both using *MEGA* version 5 [18]). Each node maintained the group assignment it had in the Mochudi data.

The simulation proceeds as follows:

1. Simulate a set of viral genetic sequences over the tree.
2. Calculate the pairwise distances between sequences using the dna.dist function in the **R** [19] package *ape* [20].
3. Record the true group-wise clustering rates 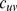 for the population of sequences for a particular threshold.
4. Sample from the observed sequences with probability (0.7, 0.6, 0.8) for the (HVL, LVL, ART) groups.
5. Estimate the adjusted (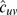) and unadjusted group-wise clustering rates for that threshold.

**Figure 1:**
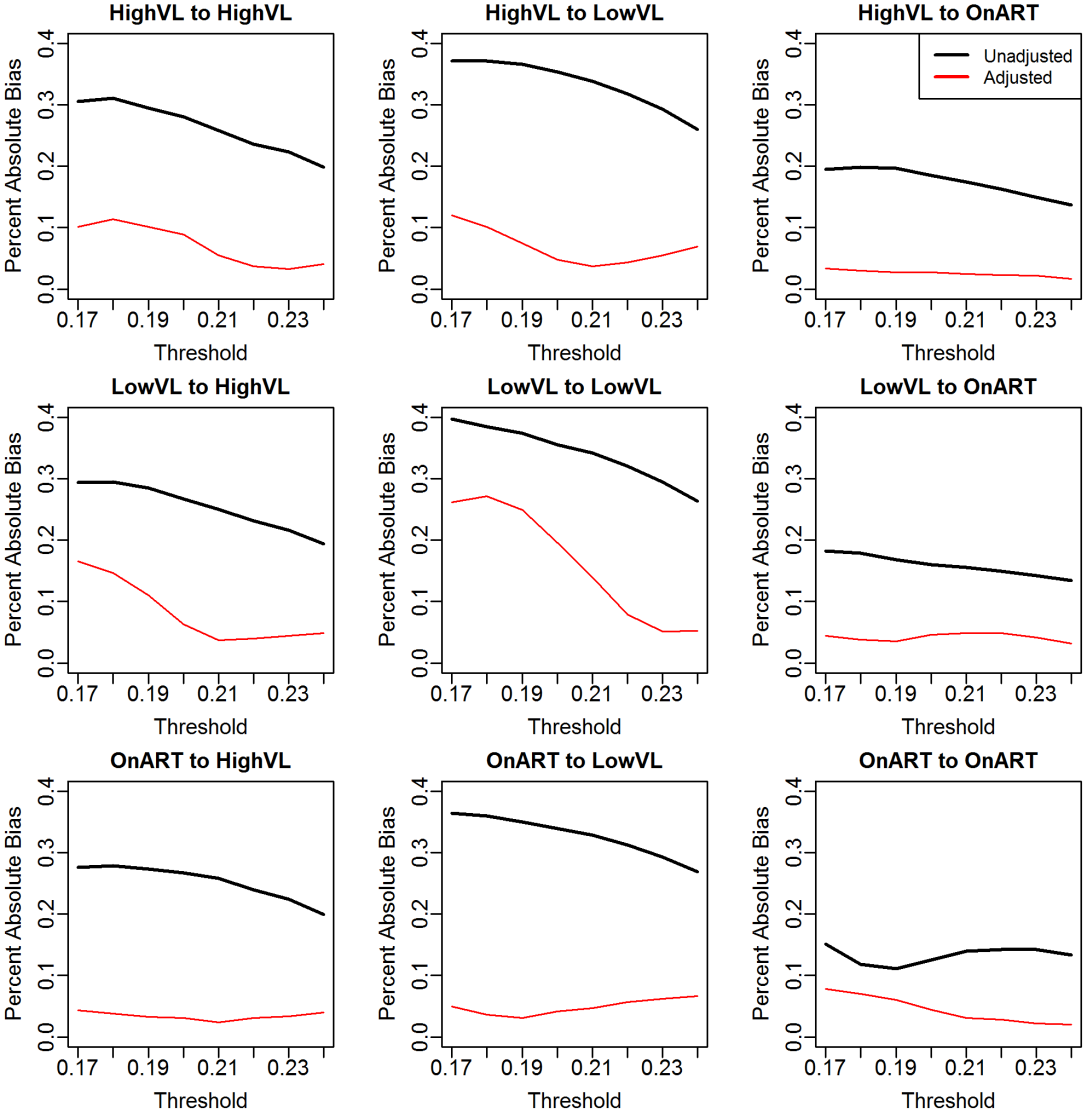
Average relative bias of adjusted (red) and unadjusted (black) estimators of group-wise linkage rates 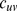 over general time-reversible evolutionary model simulations.

The threshold ranged from 0.17 to 0.24, which corresponds roughly to the 0.04th to 0.41st percentiles of the distance distribution. The expected number of links per sequence ranged from 0.04 to 0.79. We simulated 100 sets of sequences, and for each set, we simulated 100 different observed data sets for each threshold, for a total of 10,000 simulations per threshold. Figure 1 plots the mean relative bias (|estimate–truth|/truth) of the unadjusted and adjusted estimators across the range of thresholds. The unadjusted estimator has uniformly higher bias than the adjusted, and the differences in the degree of bias is often large; averaged across subpopulations (weighting by their size) and thresholds, the relative bias of 25.7% in the unadjusted analyses is reduced to 6.5% in adjusted analyses. For higher thresholds, the adjustment reduces the bias to under 5% in the majority of cases and to under 10% in all. For the lower thresholds (where linkage rates are lower), the bias in unadjusted analyses is generally greater than for higher thresholds-exceeding 35% in some cases. By contrast the bias in the adjusted analyses is below 10% in the majority of cases and below 20% in all but one. The worst performance for the adjusted analyses (low thresholds for LVL to LVL) still shows a considerable reduction in bias.

### Mochudi data analysis

In the analysis of the Mochudi household survey data, we consider three groups: HVL, LVL and ART. We observe sequences for 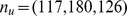 out of 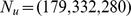 individuals in each group, yielding 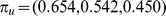. We use p-distance as our distance measure: the proportion of compared sites at which two sequences differ. Viral linkage in this analysis is defined by a p-distance below a specified value. We present the results in two ways: first, using a range of thresholds from 0.085 to 0.12 (corresponding to the 0.03rd to 0.54th percentiles), and second, focusing on a threshold of 0.1 for more detailed consideration. This latter threshold yields an overall rate of linkage of 18% within the observed sample.

**Figure 2:**
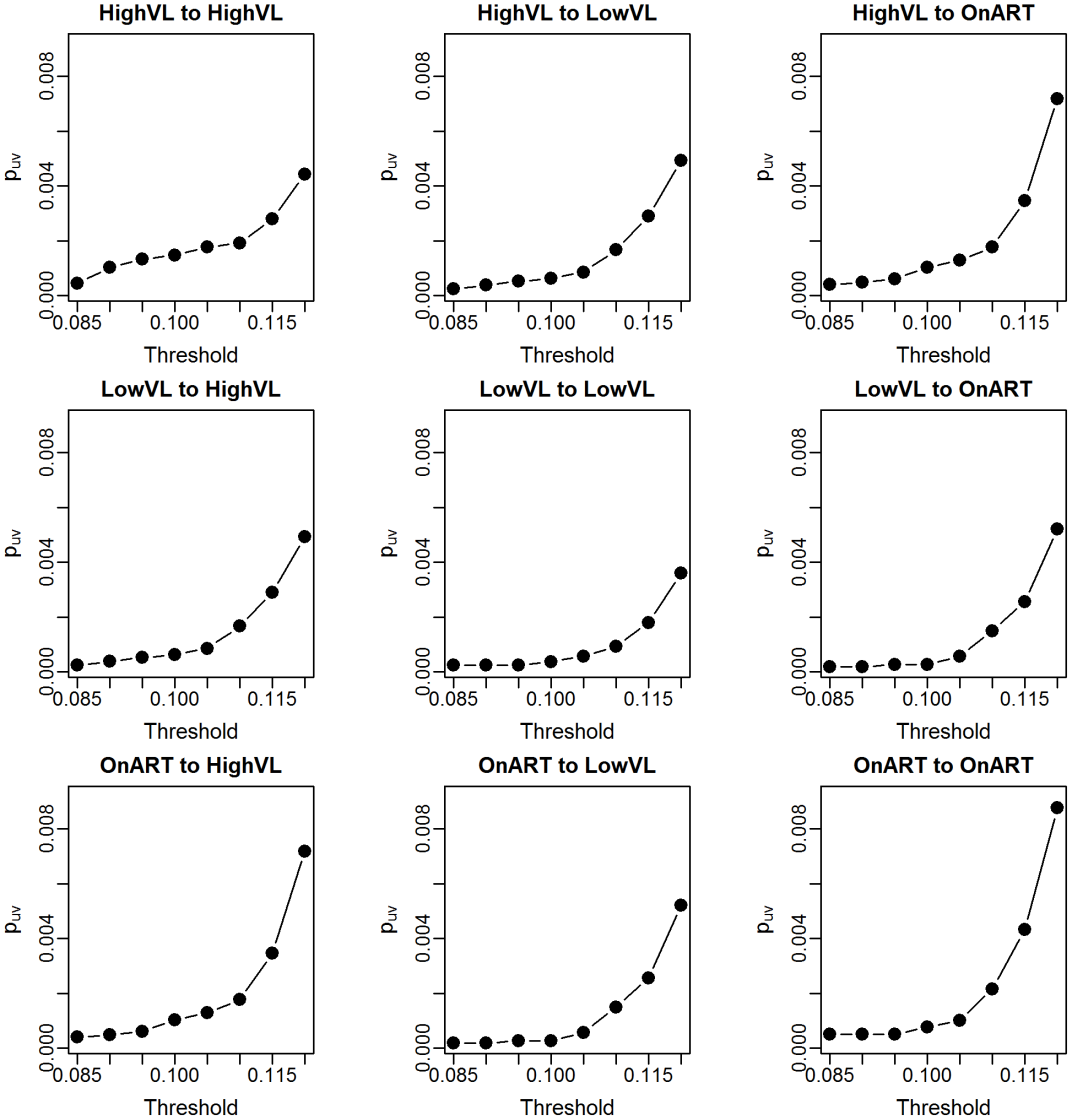
Estimates of individual-to-individual probability of linkage by groups, 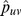, for the full Mochudi data.

Using the results for the probability of linkage between individuals, we find the 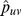 given in Figure 2 and Table 1. As one would expect, the overall probability of linkage increases with the more generous thresholds, but the pattern of relative probabilities appears to be maintained. In the table, we can see more clearly that linkage is most likely with the HVL group for all groups, while the LVL group demonstrates less linkage overall.

**Table 1:** Estimated probability of linkage between individuals of different groups, 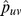, from Mochudi data.

	High VL	Low VL	On ART
High VL	1.47	0.62	1.02
Low VL		0.37	0.26
On ART			0.76

Rates given are per 1000 pairs. A link in this analysis is defined by a difference between sequences in less than 10% of available sites.

### Conditional probability of linkage

**Figure 3:**
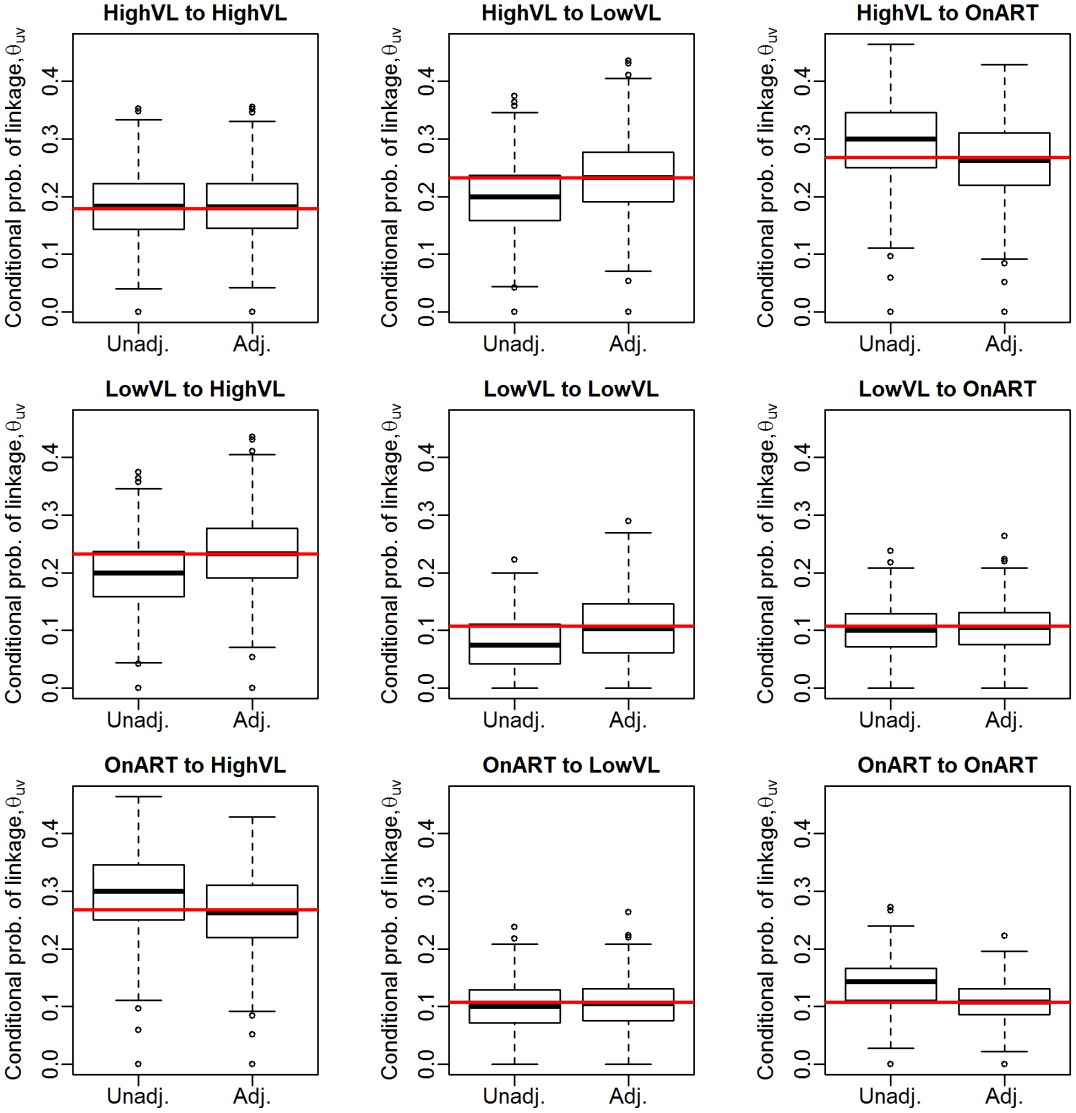
Estimates of conditional probability of linkage by groups, 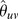, for a 70% sample of the full Mochudi data. Red line represents the “truth” as observed in the full data.

**Figure 4:**
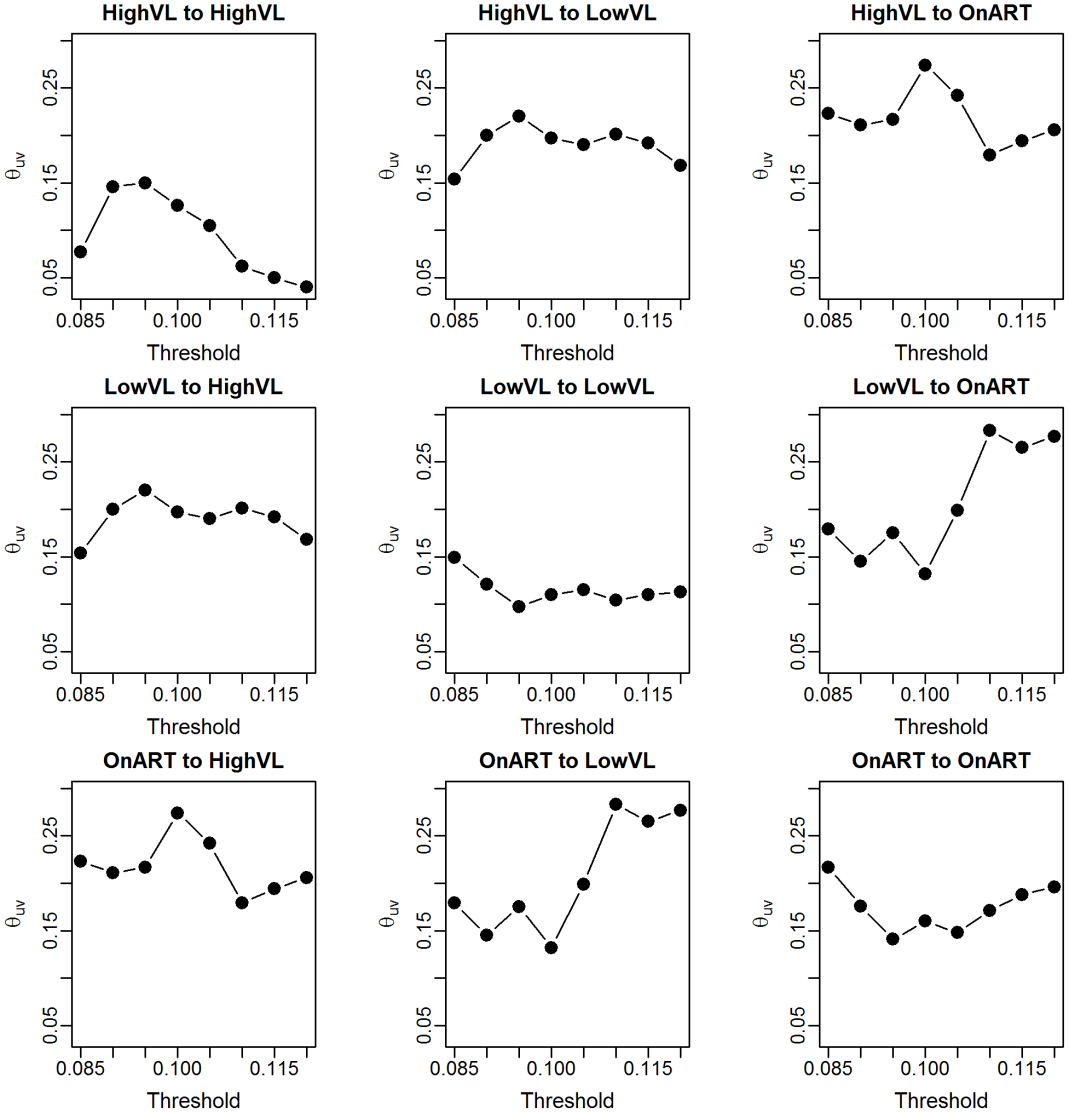
Estimates of conditional probability of linkage by groups, 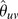, for the full Mochudi data.

We now move to estimation of the conditional probability that a linked pair are from groups 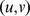, 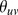. First, we examine the performance of the estimator via simulation from real data. Treating the 423 observed sequences in the Mochudi data as a full population, we sample with probability (0.7, 0.6, 0.8) from the (HVL, LVL, ART) groups. We can then record the true conditional probabilities from the full data and the unadjusted and adjusted estimates from the sampled data. Figure 3 gives the distribution of estimates of the conditional probabilities, compared against the probabilities observed in the full sample. The MLE is quite accurate, as we would expect given the generality of the results in Methods for conditional probabilities of linkage. The adjusted estimates of the conditional probabilities for the full sample are given in Figure 4 and Table 2. The relative probabilities vary more with the threshold in this case than in the individual-to-individual case, likely because the probabilities of linkage are extremely small (particularly when involving the ART group) and thus minor differences in the distribution of distances by group pair could lead to widely varying conditional probability estimates. It does appear to be most likely that a given link occurs between HVL and ART or LVL, and it is least likely to be between two LVL individuals.

**Table 2:** Estimated conditional probability of linkage between groups 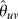 from Mochudi data.

	High VL	Low VL	On ART
High VL	0.126	0.197	0.274
Low VL		0.110	0.132
On ART			0.160

A link in this analysis is defined by a difference between sequences in less than 10% of available sites.

### Diagnostics

Before we proceed to estimate group-wise linkage rates for the Mochudi data, it is useful to examine the estimated correlation under the exchangeable model, which we will consider in development of a diagnostic tool for assessing the reliability of our methods. For the Mochudi data, we obtain a population-wide estimate of 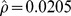; group-specific estimates are given in Table 3. Most are close to the population-level estimate, but there is some variability.

**Table 3:** Estimated correlations 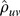 under exchangeable model by group pairing.

	High VL	Low VL	On ART
High VL	0.016	0.023	0.029
Low VL	0.018	0.022	0.023
On ART	0.020	0.012	0.021

**Figure 5:**
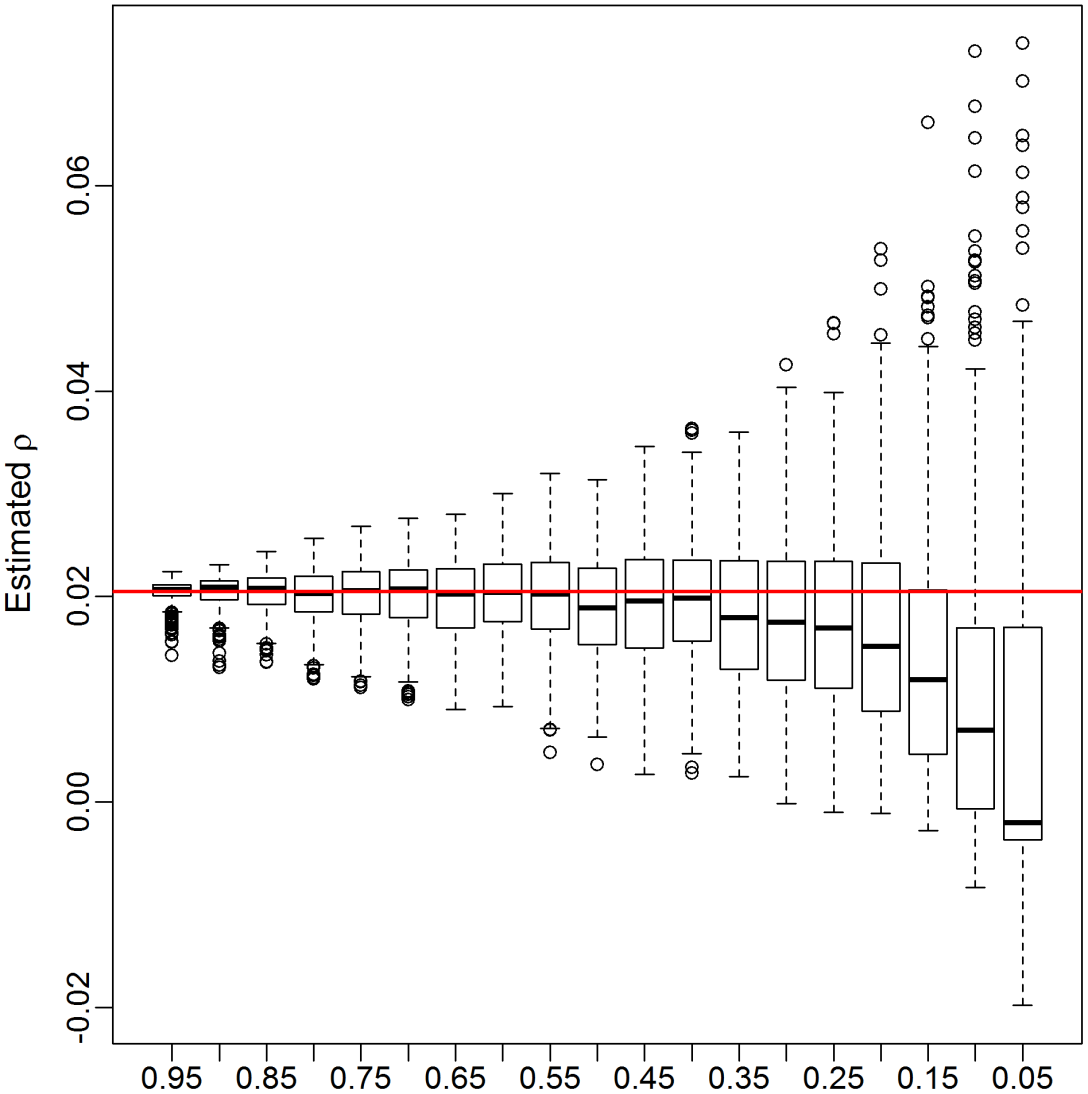
Realized values of 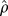 for subsamples of 5 to 95% of Mochudi data. Red line indicates the observed value of 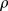 for the full data.

We can also see how the realized values of 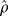 change with the sampling fraction. Figure 5 show boxplots of these realized values for subsamples of 5 to 95% of the Mochudi data. Each boxplot represents 500 samples. The estimates become increasingly variable as the sample gets smaller, but remain centered about the value of 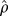 from the full data (red line) until the sample size falls below 40%, at which point the estimates decline sharply. This is likely due to an increased probability of obtaining a sample with very few observed links between the two groups. In the extreme case when no links are observed, this yields 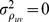 and 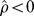, and we can expect the estimated correlation to be extremely small in cases with only a handful of links as well.

This decline in the estimated correlation, 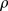, for very small samples has implications for bootstrap bias correction. We propose as a diagnostic creation of a plot similar to Figure 5 from the observed data by group. If the estimated median of 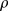 appears to remain fairly constant over a range of sampling fractions including the size of the appropriate subsample, the estimated 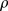 is likely to be similar to the true value, and the bootstrap bias correction should work well, since the estimated adjustment ratio described in the Methods depends upon 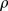 being similar across the population, observed sample, and bootstrap subsample.

### Estimation of group-wise clustering rates

**Figure 6:**
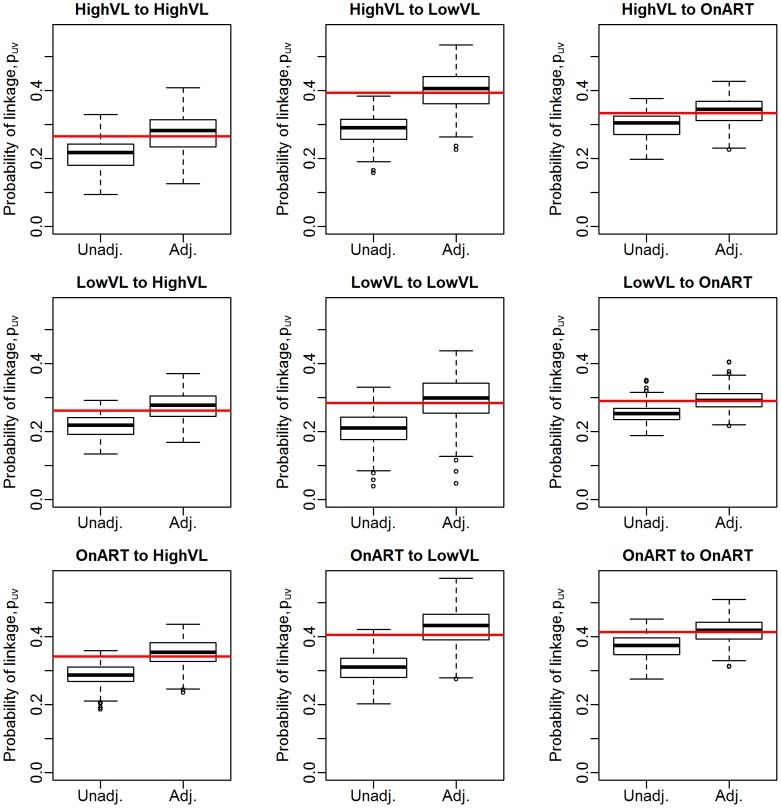
Distribution of unadjusted and bootstrap-adjusted estimators of group-wise linkage probabilities, 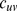 using proportionate subsampling for a 70% sample of Mochudi data.

**Figure 7:**
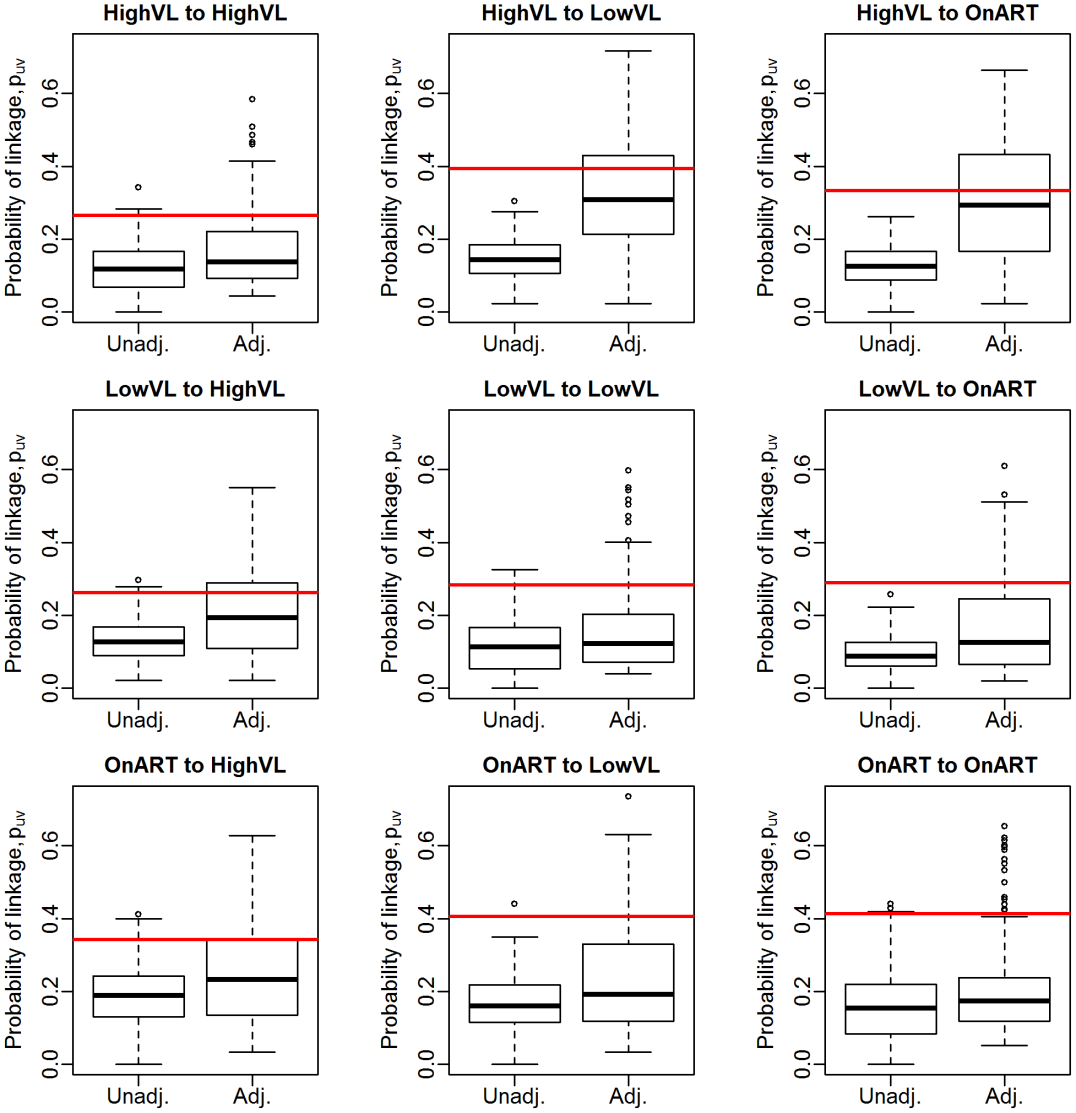
Distribution of unadjusted and bootstrap-adjusted estimators of group-wise linkage probabilities, 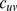 using proportionate subsampling for a 30% sample of Mochudi data.

We first assess the performance of the bootstrap estimator 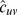 via simulation. Figures 6 and 7 show the resulting estimates for 70% and 30% samples of the observed data, respectively, including one modification: if the adjustment reduces the unadjusted estimate, we take the unadjusted value rather than the bias-adjusted value. This rule follows from knowing that the unadjusted estimate is an underestimate, implying that any reductions are likely due to very small bootstrap sample sizes or disparate correlations in the observed data and the subsample. This restriction has no effect on the 70% sample, but does impact the 30% sample substantially; we see that the bias-corrected results are not very different from the unadjusted estimates.

**Figure 8:**
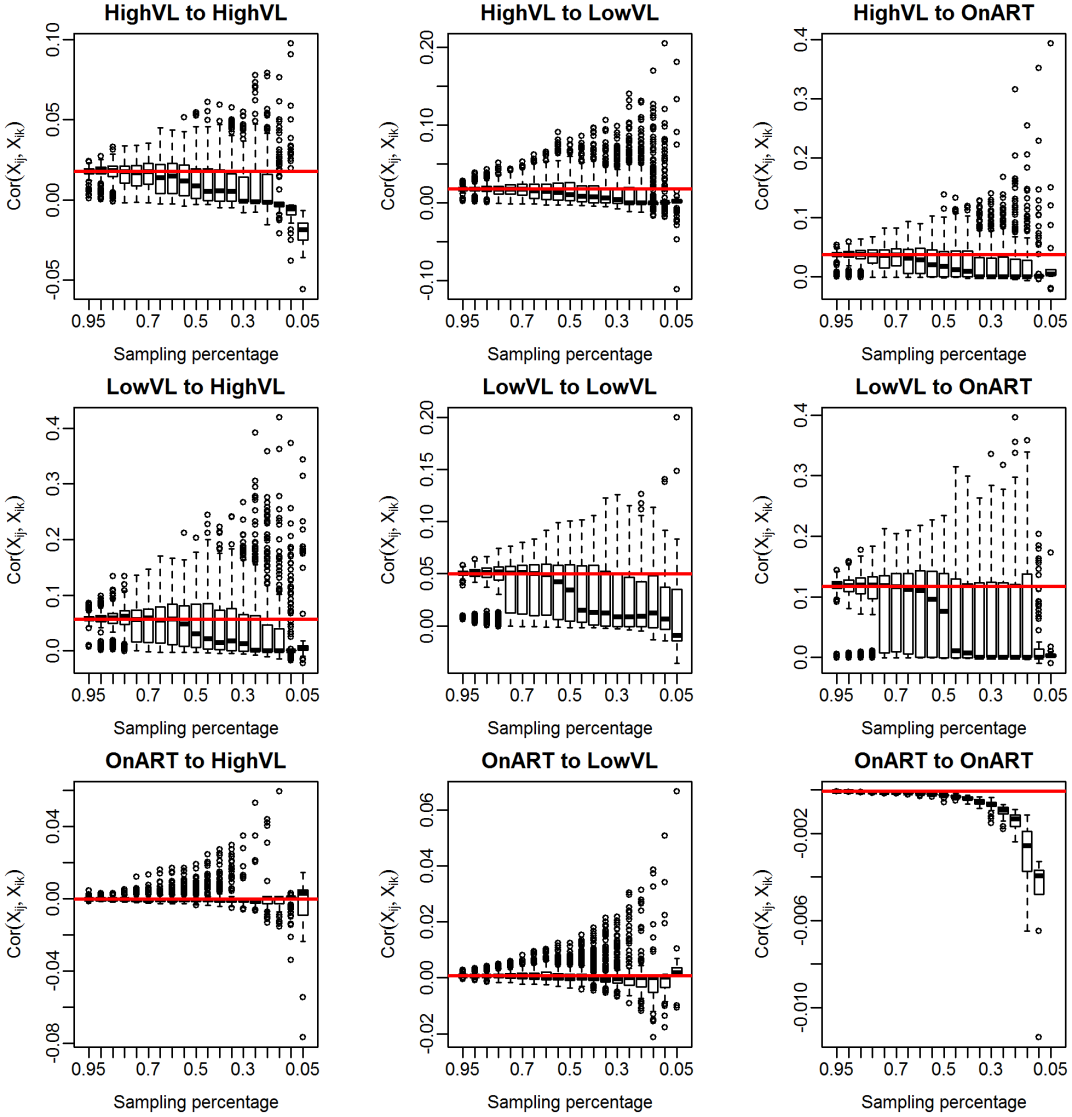
Diagnostic plots of subsample correlations by group, 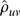.

We can now compare the estimates of 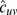 for each pair of groups using the unadjusted estimator based on observed data and using the bootstrap adjustment method. The estimated probabilities of inclusion are 65, 54, and 45% for HVL, LVL and ART, respectively. Based on the decline of 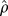 in Figure 8, we would expect that the observed correlation in the subsamples is likely to be different from the sample correlation for the LVL group, despite the theoretical possibility of obtaining the 8% subsample needed for interval subsampling. Therefore, we use interval subsampling for the HVL group, but use proportionate subsampling for the LVL and ART groups.

We calculate a confidence interval for the bootstrap adjusted estimate 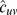 using a bootstrap quantile interval. Because the adjustment is made by taking the inverse of the bootstrap samples, the upper (lower) bound of the interval will be given by taking the 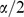 (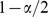) quantile of the bootstrap distribution of the ratio of the unadjusted estimate to the bootstrapped value (raised to the power of 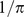 if using proportionate subsampling) and calculating 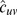 with this quantile. Simulation results show that the coverage of this interval is likely to be good as long as the sampling percentage 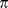 is at least 65% and may be anti-conservative if the percentage is lower. Intervals for the unadjusted estimator are found using a traditional binomial interval.

**Figure 9:**
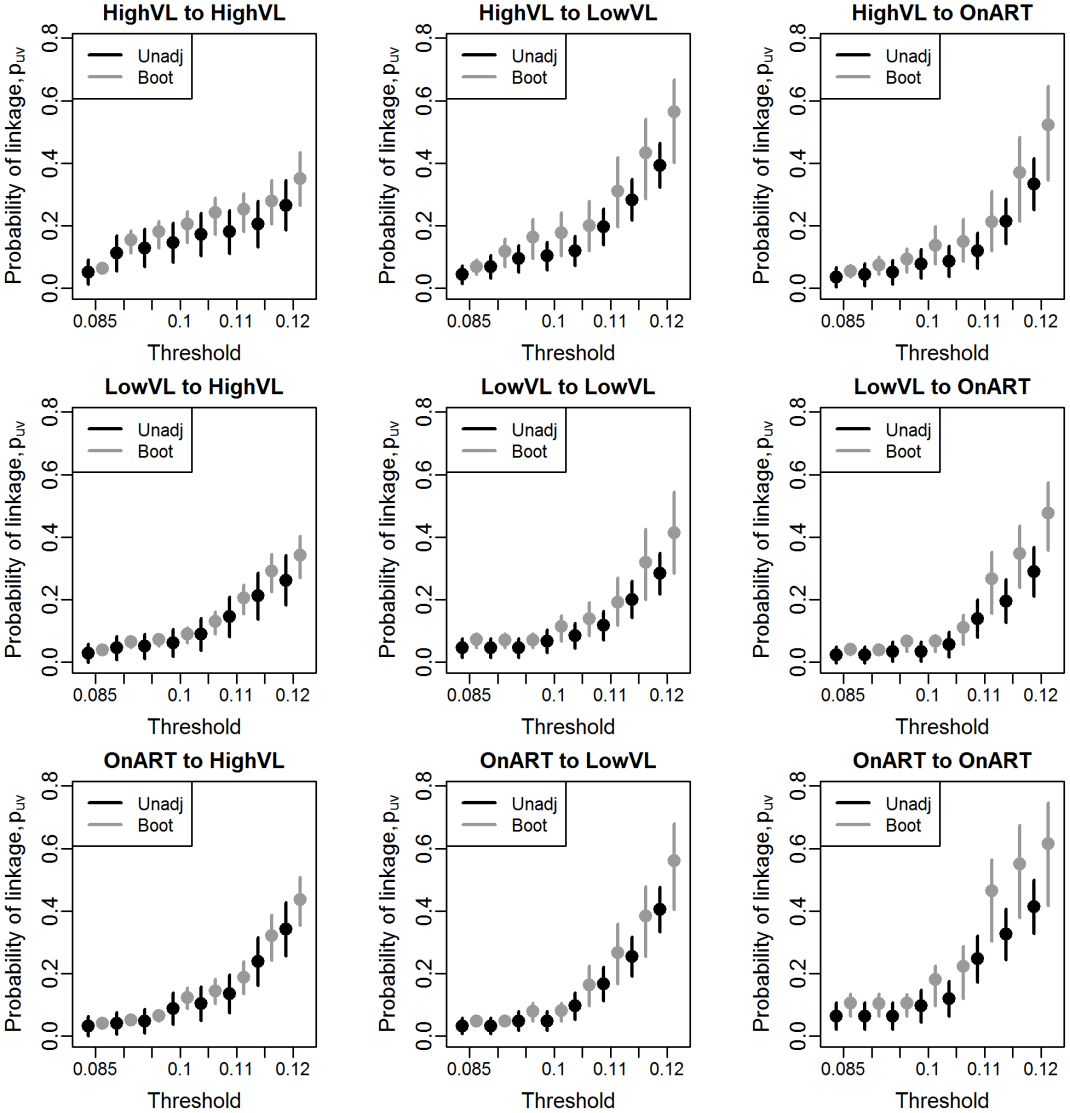
Point estimates and 95% confidence intervals for unadjusted and bootstrap-adjusted estimates of groupwise linkage rates 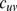 in Mochudi. The distance cutoff defining linkage between sequences ranges from 0.085 to 0.12.

We first present the results of applying the adjustment across a range of possible thresholds. As can be seen in Figure 9, the adjusted estimates are consistently higher than the unadjusted, regardless of threshold. In many cases, particularly for higher thresholds, the confidence interval for the unadjusted estimator excludes the point estimate from the bootstrap adjustment. The bootstrap quantile interval is consistently narrower than the unadjusted interval in the cases where interval subsampling was used (column 1). To see the effects of adjustment in more detail, we focus on a single cutoff of 0.1 in Table 4, where we see both estimates for the probability that a member of group A (rows) is linked with at least one member of group B (columns). The adjusted estimates range from 40 to 100% larger than those of the unadjusted estimator. The relative values of the probabilities change as a result - for example, using the unadjusted estimator, it appears that someone with high viral load is nearly twice as likely to cluster within the high viral load group as to cluster with anyone on ART. After adjustment, an HVL individual is only half again as likely to cluster within group as with ART. In this case, qualitative comparisons - specifically, the ranking of the prevalence of various combinations - remain unchanged, although it is possible in other applications that this would not be the case.

**Table 4:** Group-wise linkage rates 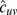 before and after adjustment for missing data.

	High VL	Low VL	On ART
	Unadj. Adj.	Unadj. Adj.	Unadj. Adj.
High VL	0.145 0.203	0.103 0.177	0.077 0.134
Low VL	0.061 0.089	0.067 0.113	0.033 0.067
On ART	0.087 0.121	0.048 0.080	0.095 0.174

A link in this analysis is defined by a difference between sequences in less than 10% of available sites.

Unlike the sequence-to-sequence or conditional probabilities of linkage, the probability of linkage from group 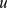 to group 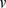 is not equal to the probability of linkage from group 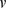 to group 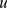. This is a function both of the sizes of the groups (if group 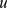 is much bigger, there are more chances for someone in group 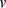 to have a link with 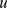 than the other way around) and of the distribution of links. Consider, for example HVL/LVL linkage; it is much more likely for someone with high viral load to link with the low viral load group than for someone with LVL to link with the HVL group. This could arise simply due to the size of the LVL group, but it is also possible that several individuals in the LVL group link with multiple individuals in the HVL group. For each such configuration, only one person in LVL is counted as having link(s) to HVL, but multiple people in HVL are counted as having link(s) to LVL.

The results for group-wise linkage rates suggest that individuals with high viral load have more links; by extension, this suggests they are involved in more transmissions or more recent transmissions. This group should include both individuals in the chronic phase of infection with poor viral suppression and recent infections [21]. Of 75 HVL with a CD4 count available, 44% had CD4 count below 250 cells/mm^3^, while only 19% of the 122 LVL individuals with a CD4 count measurement were below 250. Individuals on ART are most likely to link within their own group, suggesting links from older transmissions in which both individuals have progressed to the point of needing treatment. Individuals in the low viral load group link relatively little. Without information on the relative timing of infections (such as will be available from the BCPP), we cannot make inference about transmission contributions. However, taken together, these results are at least consistent with the hypothesis that those with low viral load, either from natural suppression or treatment, are not transmitting as efficiently as those with high viral load. Data on prevalence and incident cases collected over time will permit more formal testing of this hypothesis.

### Choice of distance measure

Many different methods are available for calculating distances between genetic sequences [22]. As the definition of linkage is a simple threshold on the distance between sequences, distance models that give different results could result in rate estimates that vary widely.

We compared four different distance calculation methods on the set of 423 sequences from the first year household survey in Mochudi, Botswana. The methods compared were:

- pdist: proportion of compared sites at which sequences differ (denominator varies by pair due to pairwise deletion)
- mcl: maximum composite likelihood
- jc: Jukes-Cantor model
- t3: Tamura 3-parameter model

Codon positions included were 1st+2nd+3rd+Noncoding. All positions with less than 95% site coverage were eliminated. That is, fewer than 5% alignment gaps, missing data, and ambiguous bases were allowed at any position. There were a total of 1050 positions in the final dataset. Analyses of uncorrected pairwise distances and corrected by different evolutionary models were conducted in *MEGA* version 5 [18].

The scale or mean of the distance distribution might be expected to vary over the methods used to calculate the distances. To reduce this source of variability, we treat the threshold for linkage as a quantile of the distribution (i.e., the bottom 10% of distances cluster), thereby ensuring that measures that maintain the same ranking of distances provide equivalent results. Table 5 gives the Spearman rank correlation matrix of the five methods listed above. As the Spearman correlation considers only ranks, any measures that are equivalent up to a monotonic increasing transformation will have a correlation of 1.

**Table 5:** Spearman rank correlation of four distance methods.

	pdist	mcl	jc	t3
pdist	1.0000	0.9990	1.0000	0.9992
mcl	0.9990	1.0000	0.9990	0.9964
jc	1.0000	0.9990	1.0000	0.9992
t3	0.9992	0.9964	0.9992	1.0000

All four methods used have nearly perfect correlation, indicating that applying the analysis methods described here with a quantile-based cutoff will result in nearly identical results regardless of the distance model used.

## Discussion

Genetic linkage analyses have been useful in making inferences about important HIV epidemic drivers, including the impact of acutely or recently infected subjects [1], [2], [4]. Application of these methods to community randomized trials of HIV prevention interventions such as the cluster randomized trial of HIV prevention in Botswana [10], [23] may be useful not only for this purpose but also to provide information regarding the subpopulations in which these interventions are succeeding or failing. For example if newly infected subjects in communities randomized to the intervention cluster only with viruses that infect people living outside the community, this knowledge would imply that the intervention is succeeding in stopping transmission within communities. The implications for the success of the intervention are very different from the setting in which newly infected cases are in fact being infected with viruses circulating within communities. For the latter, it is important to know what subgroups contribute most to onward transmission of virus, whether these subgroups be defined by plasma HIV levels, ART treatment status, or demographic or behavioral factors. All such analyses, however, are very much impacted by potentially informatively missing data. This paper proposes methods to adjust for such biases.

Our methods adjust for the presence of missing viral sequences in estimates of viral linkage rates under the assumption that sequences are missing at random conditional on group membership. We show that we can consistently estimate the probability that two sequences are linked without adjustment for missing data, and can consistently estimate conditional probabilities of linkage between two sequences from a pair of groups given the existence of a link via a minor adjustment using the (known) sizes of the groups in the population. In settings where it is reasonable to assume that the linkage status of pairs of sequences are all independent conditional on group, the estimator presented for estimation of group-wise linkage probabilities under independence is in fact the MLE and provides an exact solution. This assumption might be reasonable in investigations of airborne pathogens, or in settings with sparse sampling.

For settings in which the assumption of independence is not reasonable, we propose a bootstrap resampling approach to adjust for the bias in the unadjusted estimator. If linkage indicators are exchangeably correlated or if their correlations can reasonably be modeled as functions only of individual effects (a random-effects type model) and we can use interval subsampling, then the resampling method can adequately adjust for bias. When using proportionate subsampling under the random-effects model the bootstrap may under-correct, but the resulting estimates are still preferable to those provided by unadjusted estimators. We note that departures from the assumption of a random effects structure in the correlation would arise if the probability of linkage depended not only on the individual characteristics of sequences and the people infected with them, but also, in unspecified ways, on the interactions between these characteristics. In such cases, unbiased adjustment for missing data is not possible, because such departures would imply that unobserved linkages followed a different process from those that are observed. Even in this case, however, it would be useful to employ our methods, because they at least provide estimates that are valid under much broader assumptions than in the case for unadjusted analyses and they demonstrate the effect of the broadening of assumptions on results. Large changes in estimates provide caution against overinterpretation of results.

Furthermore, our simulation results using the Mochudi data suggest that the adjustment may be adequate in some realistic settings where the assumption of the random effects structure may not hold perfectly. To provide guidance on appropriate usage of the method, we propose a diagnostic tool that provides assessment of the likely reliability of the bootstrap resampling approach to adjust estimates of clustering rates.

The choice of the threshold defining linkage will vary broadly with the goal of analysis and methods of data collection. This choice is critical to any linkage analysis, and sensitivity to the choice of threshold should be examined. The methods developed here can be applied to any threshold or range of thresholds in order to obtain linkage rate estimates that are adjusted for the presence of missing data. Considering adjusted results for a range of thresholds will permit more reliable comparisons between groups and between thresholds.

Although the groups of interest for linkage and those of relevance for the missingness model were the same in our example, this condition is not required. A more general missingness model could be formed by creating a partition into subgroups such that pairs of observations are missing at random given subgroup membership. Our method would then proceed by first estimating linkage rates for each of these subgroups, and then aggregating across them to obtain the estimates for the groups of interest (as suggested in Methods). As an example, to address our fundamental goal of estimating the relative contributions of within-community and outside-community partners to new infections, we would include community as one of the variables that defines our groups. We might, for example, define groups as community by sex by age category, for example. Given age- and sex-specific prevalence estimates for each community, we can adjust for missing data within these categories, and then aggregate to the level of community, yielding estimates of the proportion of individuals in community 1 who cluster with community 2 and vice versa, as well as the proportion who cluster within their own communities. Such an analysis will provide an indication of the relative force of infection from within versus outside the community, especially if we have separate groups for incident infections. The methods can also be extended to allow the model for missingness to depend on continuous-valued variables.

The approach discussed here is not restricted to linkage indicators defined by a pairwise distance cutoff. The rate of occurrence of any feature of interest that can be coded as an indicator variable for each pair of sequences can also be estimated with adjustment for missing data. Beyond the change in the definition of a link, the application of the method is identical.

The bootstrap method described here is similar in spirit to inverse-probability weighting in that adjustment for bias makes use of information on the probability of observation to estimate a scaling factor. In our setting, however, it is not possible to express the weight in closed form because of the complex correlation structure induced by the vagaries of HIV evolution and of patterns of viral transmission.
